# How landscape factors relate to biodiversity-economic performance in an Estonian grassland-rich region

**DOI:** 10.1007/s00267-026-02533-x

**Published:** 2026-07-07

**Authors:** Takamasa Nishizawa, Johannes Schuler, Miguel Villoslada, Claudia Bethwell, Michael Glemnitz, Maaria Semm, Monika Suškevičs, Kalev Sepp, Rando Värnik, Florian Kestel, Meelis Ots, Peter Zander, Zander Venter, Sandra Uthes

**Affiliations:** 1 Leibniz Centre for Agricultural Landscape Research (ZALF), Müncheberg, Germany; 2 Department of Biology, School of Experimental Sciences and Technology, Rey Juan Carlos University, Madrid, Spain; 3 Geography Department, Humboldt-Universität zu Berlin, Berlin, Germany; 4 Institute of Agricultural and Environmental Sciences, Chair of Environmental Protection and Landscape Management, Estonian University of Life Sciences, Tartu, Estonia; 5 Institute of Agriculture and Environment, Chair of Rural Economics, Estonian University of Life Sciences, Tartu, Estonia; 6 Institute of Veterinary Medicine and Animal Science, Chair of Animal Nutrition, Estonian University of Life Sciences, Tartu, Estonia; 7 Norwegian Institute for Nature Research – NINA, Oslo, Norway

**Keywords:** semi-natural grassland, biodiversity conservation, landscape factors, biodiversity-economic performance, remote sensing

## Abstract

Halting biodiversity loss on semi-natural grassland has become a key priority of agri-environmental policies in Estonia. However, to what extent current schemes are sufficient to achieve biodiversity targets and the role of landscape factors is still insufficiently understood in this context. This study explored trade-offs between profitability and biodiversity conservation across heterogeneous Estonian agricultural landscapes to support the development of economically effective grassland conservation schemes. To this end, we selected twelve ‘landscape windows’ in a grassland-rich region that represent a gradient of several biodiversity-related landscape factors (semi-natural grassland share, yield potential and landscape complexity). Profitability was assessed using total gross margins derived from a bio-economic model, while biodiversity was evaluated through habitat values for different bird species, based on model outputs. We found that landscapes with higher complexity, which currently maintain higher biodiversity levels, had a corresponding lower farm profitability. In particular, landscape windows dominated by semi-natural grasslands with low grass yields showed the lowest profitability, largely due to limited capacity for feeding beef cattle. In contrast, arable land-dominated landscapes (i.e. those with higher yield potential) demonstrated considerable potential for enhancing biodiversity outcomes while minimising profitability losses. Therefore, conservation policy faces two main challenges: 1. preserving landscapes with high biodiversity value against further decline in profitability, 2. highlighting the need to explore and design biodiversity measures in arable-dominated landscapes that could improve biodiversity outcomes while limiting impacts on farm profitability. This study illustrates that landscape factors deserve greater attention in conservation planning.

## Introduction

Agricultural intensification and simplification of landscape structures have negatively affected biodiversity in the past decades (Marja et al., 2022). In response, various biodiversity-enhancing measures aiming at reversing biodiversity decline, for example, European Union (EU) agri-environment schemes (AES), have been introduced, which may partly compensate for biodiversity losses or even enhance biodiversity, yet have often been criticized for not achieving long-term impacts and failing to reach biodiversity targets (Brown et al., 2019; Uthes and Matzdorf, 2012). The lack of success can often be attributed to their isolated focus on land cover properties while neglecting landscape contexts and farm characteristics (Paulus et al., 2022). Dauber et al. (2010) and Rowe et al. (2013) have shown that the effectiveness of biodiversity measures can vary depending on several factors, including the surrounding landscape type, the type of land use being replaced (i.e. indirect land use change), the species groups targeted and how the measures are managed.

In contrast, a holistic strategy towards conservation management in multifunctional agricultural landscapes is purposefully arranged along a management intensity gradient and contributes to creating effective ecological networks to protect species and genetic diversity while ensuring forage production and other provisioning ecosystem services (ES) (e.g. Bennett et al., 2021; Manning et al., 2018). From an economic perspective, biodiversity-enhancing measures should ideally be implemented in a cost-effective way so that the biodiversity gains in relation to incurred farm costs are maximised (Wätzold et al., 2008; Wätzold & Schwerdtner, 2005), thereby searching for win-win situations for nature, farms and society. A key challenge, however, is that land-use decisions are made at the individual farm scale (Méité et al., 2024), focusing on private values, while biodiversity and ES, which offer multiple benefits to society, emerge at the landscape scale (e.g. Armsworth et al., 2012). Yet under supporting conditions, depending on design, spatial arrangement and a targeted implementation, also ecological compensation measures and biodiversity-promoting in-field measures can enhance biodiversity on farmland (Stoeckli et al., 2017).

Empirical results and meta-analysis studies show that greater landscape complexity is associated with higher biodiversity levels (Marja et al., 2022), although fragmentation and homogenisation due to habitat losses may reduce biodiversity and ecosystem functions (Liu et al., 2018). In contrast, AES effectiveness tends to be higher in simple landscapes compared to more complex ones with a large proportion and diversity of semi-natural areas (Marja et al., 2022). This is because AES measures implemented in simple landscapes can readily create ecological contrast in floral resources compared to the surrounding floral-poor farmland (Scheper et al., 2013). The intermediate landscape complexity hypothesis posits that local conservation management has the highest landscape-moderated effectiveness in structurally simple but not in extremely simplified or in complex landscapes (Tscharntke et al., 2012). In ecology, landscape complexity is measured with indicators for landscape composition, landscape configuration and the resulting connectivity within habitats.

This paper investigates the role of different landscape factors on the performance of biodiversity provision, considering economic performance (i.e. biodiversity provision relative to economic performance) in an Estonian grassland-abundant region. Specifically, we explore how relative biodiversity–economic performance differs depending on varying landscape characteristics. In this study, landscape factors are treated as structural characteristics that define landscape configurations and condition the shape of the profitability–biodiversity trade-off, rather than as independent causal drivers of either outcome. To explore the role of different landscape factors for this study, we employ a ‘landscape window’ approach (Kay et al., 2017, 2018; Naaf et al., 2021), in which each landscape window represents different characteristics of landscapes and use bio-economic farm modelling (Nishizawa et al., 2024; Schuler et al., 2013; Uthes et al., 2010) and ecological modelling to assess the habitat value of agricultural land for indicator bird species of farmland (Brandt and Glemnitz, 2014; Glemnitz et al., 2015) to simulate how biodiversity provision and profitability vary among these landscape windows.

With our findings, we aim to contribute to a better understanding of the role of landscape factors on biodiversity provision to inform the decision-making of land managers and policy implications with regard to economically efficient grassland conservation measures.

## Material and methods

### Case Study Region

**Fig. 1:**
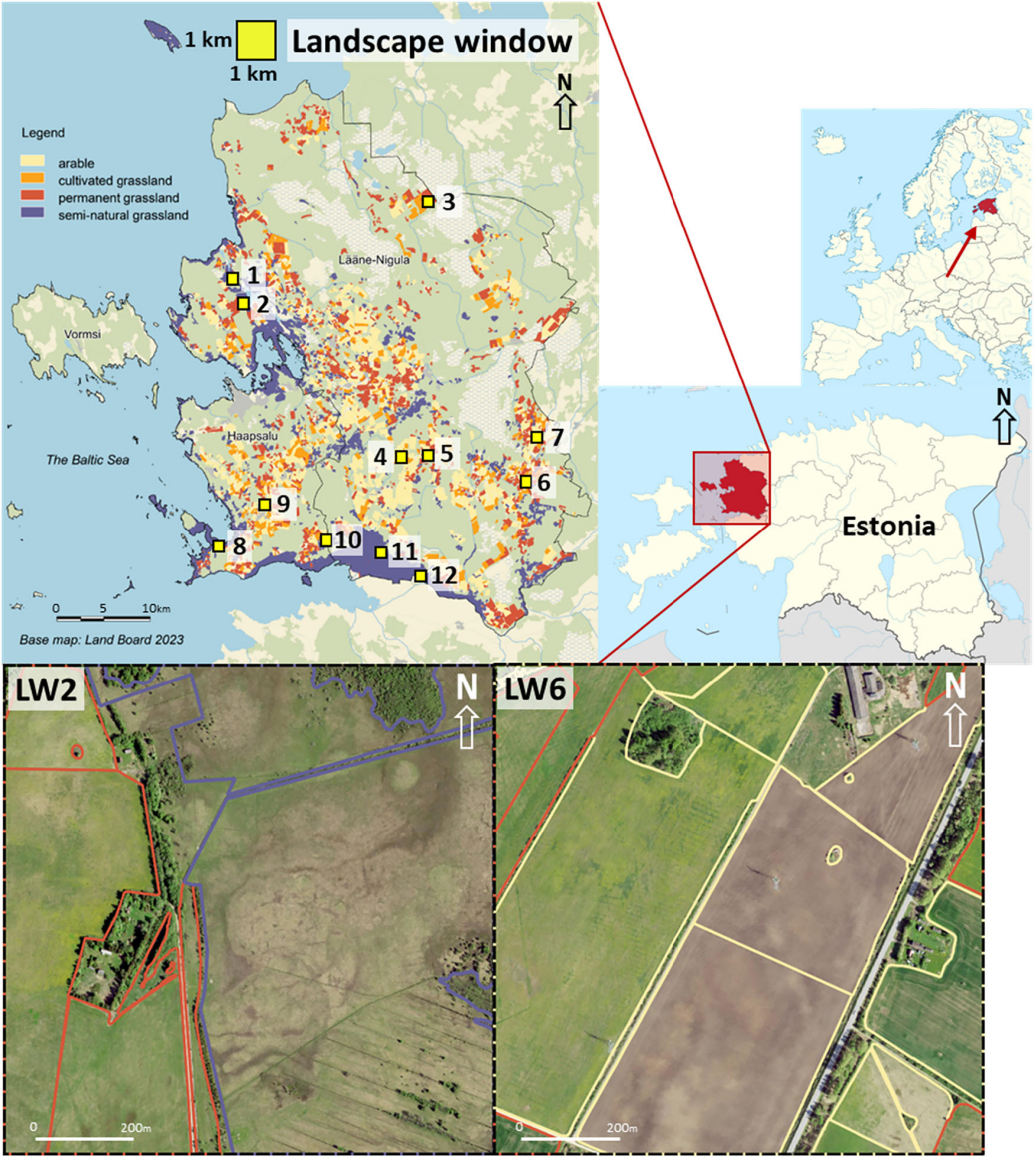
(above) Study area (Lääne County, Estonia) and the location of the selected twelve landscape windows (LW). (below) two exemplary landscape windows with semi-natural grassland (SNGL) (LW2-left) and without SNGL (LW6-right). The areas bordered by blue, red and yellow lines are SNGL, permanent grassland and arable land, respectively. Source of background imagery: Republic of Estonia Land and Spatial Development Board (https://geoportaal.maaamet.ee/)

Our case study region is the coastal Lääne County (2383 km^2^) (Fig. 1) in western Estonia, bordering the Baltic Sea with an average annual temperature ranging from 6.1°C to 7.8 °C and an annual precipitation of 500 to 700 mm. The total number of farm holdings with more than one hectare is 476 (average farm size: 78 ha). Most farms combine crop cultivation with livestock production, primarily beef cattle for meat production, while some focus exclusively on crop production.

The landscape of Lääne County is topographically flat but diverse with respect to grassland use. According to data from multiple sources—including the Estonian Nature Information System (2022)- approximately 43% of the agricultural land, corresponding to approximately 43,000 hectares, is used as grassland. Of this, 70% belongs to semi-natural grassland (SNGL) and 30% to permanent grassland. In this study, SNGL refers to grasslands established naturally from wild grasses and herbs that have not been subject to ploughing, seeding with cultivated plants, or fertilisation—typically more than 30 years (The Estonian Environmental Board, 2020). Diverse types of habitats are seen on SNGL, which are maintained by grazing and mowing. SNGL is clearly distinguished from fallow lands, where cultivation is only temporarily taken out, hence naturally established plant communities are insufficient for it to be categorised as SNGL. SNGL must meet additional criteria, including the presence of a natural biota shaped by long-term human management practices such as mowing and grazing. This natural biota supports a variety of grassland habitats protected under the EU Natura 2000 network. Biomass production on these types of grassland is not only used for livestock production but also for a local biomass heating plant or exporting abroad as part of efforts to prevent the abandonment of management. The remaining area (57%) is used as arable land, where cultivated grassland (i.e. field grass), winter wheat, spring barley and winter rapeseed are mainly cultivated. Permanent grassland refers to more intensively managed grassland with fertilisation and frequent cuttings, but not ploughed for at least five consecutive years and then re-established with new grass seeds. It is used for grazing and mowing up to three times a year. In addition to producing hay, the cut grass on this site is also used for producing silage or just mulched (i.e. it is left on the field).

Lääne County serves as a relevant case area for this study, as the region actively promotes grassland use due to its potential socio-ecological benefits, including preserving biodiversity and cultural heritage. Such efforts require introducing grassland-targeted policy measures that consider various landscape features for more economically and ecologically effective grassland management.

### Selection of Landscape Windows

We selected 12 landscape windows within Lääne County. The landscape window approach was used in accordance with Naaf et al. (2021) to represent typical examples of the agricultural landscape in the case study region, whereby the two-stage selection process and selection criteria are summarised in the Supplementary Material 1.

According to the selection process and the selection criteria, each landscape window contained at least 80% agricultural land (arable land, permanent meadows, or semi-natural meadows). This threshold was set to ensure that the key analyses by economic and ecological modelling relate to agricultural land, for which the modelling methods of the bio-economic farm modelling and the ecological modelling to assess the habitat value of agricultural land for indicator bird species of farmland are designed for this type of land and were used accordingly to achieve the research objectives of this study.

Within these windows, we also mapped landscape structural elements, defined as individual trees, tree rows, stone heaps and stone walls. Geospatial data, including land cover classes and green infrastructure features, were obtained from the Estonian ecosystem types Basemap (Helm et al., 2020) and the Estonian Base Map, produced by the Estonian Land and Spatial Development Board. These landscape windows represented thus contrasting areas of the agricultural landscape in the case study region in terms of the presence of semi-natural grassland, with half of them containing semi-natural grassland and the other half not. Accordingly, the half with the high proportion of semi-natural grassland has predominantly no or only a small proportion of arable land. Taking into account the other characteristics of land use cover, there is additionally a gradient in landscape complexity, which was calculated more precisely using the Shannon index and incorporated into the analyses (see below).

The representative sample of landscape windows was initially obtained using a random procedure. Using QGIS, we generated 40 random points within areas classified as agricultural land in Lääne County. Each point defined the centroid of a 1 km² window. We then calculated the land cover composition within each window and retained only those with ≥80% agricultural land. A minimum distance of 2 km between window centroids was enforced to reduce spatial autocorrelation. To ensure a sufficient gradient in the presence of landscape structural elements and to avoid spatial overlap, some window locations were slightly adjusted—i.e. repositioned within nearby suitable areas—to meet the selection criteria. The final selection included 12 representative, non-overlapping windows (6 with landscape structural elements, 6 without), spatially distributed across the county.

To assess potential spatial autocorrelation among the selected landscape windows, we calculated Moran’s I for the landscape complexity index using a k-nearest-neighbors spatial weights matrix (k = 4) and 9999 Monte Carlo permutations. The test indicated no significant spatial autocorrelation (I = −0.141, *p* = 0.83), supporting the spatial independence of the windows.

The resulting 12 landscape windows (1 km grid cells, Fig. 1) represent different characteristics of landscape elements, soil quality and the share of SNGL. In Fig. 1, two exemplary images of the landscapes are shown. Table 1 Error! Reference source not found. shows the observed land cover types and landscape elements across the landscape windows. Table 2 presents the types of semi-natural habitats found in the 6 landscapes with SNGL across the landscape windows.

**Table 1:** Characteristics of the selected landscape windows

	Landscape window											
Land cover/features	1	2	3	4	5	6	7	8	9	10	11	12
Agricultural area (ha)	92.88	92.69	93.13	90.32	85.39	83.28	82.67	84.3	92.22	90.43	96.36	92.12
SNGL (%)	82%	71%	0%	0%	0%	0%	0%	96%	0%	18%	100%	100%
Permanent grassland (%)	0%	29%	25%	0%	40%	23%	17%	4%	3%	12%	0%	0%
Arable land (%)	18%	0%	75%	100%	60%	77%	83%	0%	97%	70%	0%	0%
Singular trees (number)	20	19	1	8	10	39	7	20	18	37	9	3
Tree rows (m)	642	1060	1292	398	3177	1413	176	2397	377	233	897	1589
Stone heaps (number)	3	11	0	0	0	0	4	2	3	0	4	10
Stone walls (m)	40	0	0	0	0	0	0	0	0	0	89	31

*SNGL* semi-natural grassland

**Table 2:** Area in hectares of different semi-natural habitats across the landscape windows. Only landscape windows containing SNGL are listed; all others are excluded

		Landscape window with SNGL					
Code^a^	Name of habitats	1	2	8	10	11	12
1630	Boreal Baltic coastal meadows	13.31	0.00	78.45	0.00	0.00	0.00
6280	Nordic alvar and Precambrian calcareous flatrocks	0.00	0.00	0.10	0.00	0.00	0.00
9070	Fennoscandian wooded pastures	0.00	2.38	0.00	14.52	0.00	0.00
6450	Northern boreal alluvial meadows	0.00	0.00	0.00	0.00	96.35	92.12
7230	Alkaline fens	62.68	63.49	0.00	0.00	0.00	0.00
6510	Lowland hay meadows	0.00	0.00	0.00	2.08	0.00	0.00
6270	Fennoscandian lowland species-rich dry to mesic grasslands	0.00	0.00	1.98	0.00	0.00	0.00

^a^EU habitat types according to the EU Habitats Directive.

### Landscape Factors

Based on a set of collected data of the selected landscape windows, we first define three landscape factors that represent the characteristics of each landscape window: A SNGL share, yield potential and complexity of landscapes. We then investigate how each of these factors is related to profitability and biodiversity level and finally, relative biodiversity–economic performance within a landscape window.

The share of SNGL and arable land in each landscape window was determined using GIS analysis. In this study, the share of arable land represents yield potential (i.e. higher arable land share, higher yield potential). To aggregate the landscape factors that describe the landscape’s potential for supporting a high biodiversity value, a composite landscape complexity index was developed. We assume that landscapes are more complex when they contain a greater number of structural elements. Therefore, this index combines SNGL share, a Shannon index for crop diversity (Uthes et al., 2020) and the share of structural elements in landscapes of each landscape window according to the equations (1-1), for landscape containing arable land and (1-2) for landscapes without arable land, respectively:

1-1 $$\begin{array}{cc}{{\rm{For}}}\; {{\rm{a}}}\; {{\rm{landscape}}}\; {{\rm{window}}}\; {{\rm{with}}}\; {{\rm{arable}}}\; {{\rm{land}}} & {{LC}}_{i}=({{SGNL}}_{i}+{h}_{i}+{{LE}}_{i})/3\end{array}$$

1-2 $$\begin{array}{cc}(1-2){{\rm{For}}}\; {{\rm{a}}}\; {{\rm{landscape}}}\; {{\rm{window}}}\; {{\rm{with}}}\; {{\rm{only}}}\; {{\rm{grassland}}} & {{LC}}_{i}=({{SGNL}}_{i}+{{LE}}_{i})/2\end{array}$$

where SNGL, h and LE represent a SNGL share, a Shannon index for crop diversity on arable land and the share of structural elements in landscapes, respectively.

Due to the unavailability of data on cropping patterns on arable land within each landscape window, the Shannon index in this formula was calculated based on the simulated cropping pattern, using the model described in Section 2.5. It was normalised by dividing the calculated Shannon index by the maximum possible Shannon value (Breitschuh et al. 2008), given the full set of crop types available in the simulation model, resulting in values ranging from 0 to 1.

For the landscape structure index, individual structural elements (number of individual trees, length of tree rows, number of stone heaps and stone walls) were first quantified for each landscape window and then combined into a single index, representing the relative abundance of structural features within each window. It was also normalised to a 0–1 range by dividing each value by the maximum value observed across all landscape windows. This way, all components of the landscape complexity index were normalised to a 0–1 range prior to aggregation. Please note that it is beyond the scope of this study to disentangle the ecological effects of different structural elements, which may contribute differently to ecological diversity.

For profitability, we look at the gross margin that can be generated from the whole farming activities within each landscape window. For biodiversity, we use the habitat value scores (explained in detail in Section 2.6). For relative biodiversity–economic performance, we look at the number of habitat value percentage points that can be obtained by one euro of the gross margin gained per hectare of land available for agriculture within a landscape window (i.e. relative biodiversity–economic performance indicates a relative biodiversity score given an obtained gross margin).

### Hypothesised Outcomes

Error! Reference source not found. outlines the hypothesised relationships that we investigate between the three landscape factors and three outcome dimensions: profitability, biodiversity and relative biodiversity–economic performance.

**Fig. 2:**
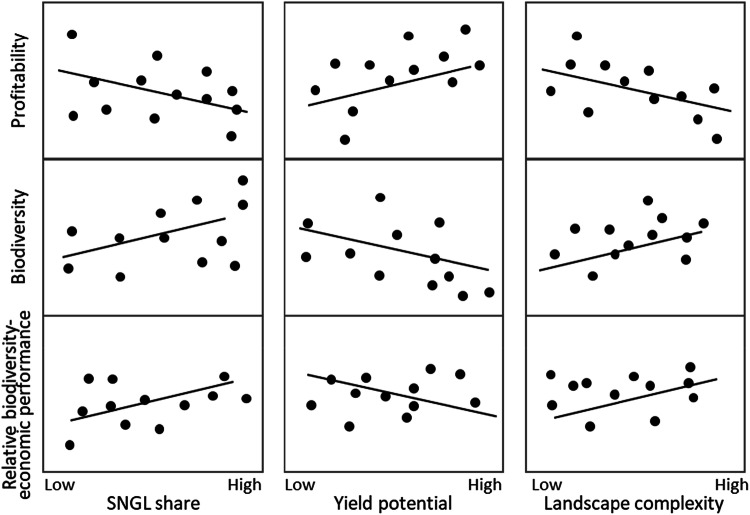
Hypothesised relationships between three outcome dimensions: profitability, biodiversity and relative biodiversity–economic performance. SNGL semi-natural grassland. Please note that hypotheses concern the direction (positive, negative) of the relationships, not their strength (slope coefficient)

First, we hypothesise that profitability (i.e. the farmers’ income that can be generated from farm activities within a landscape window) is negatively associated with a share of SNGL (Error! Reference source not found., top-left) and landscape complexity (Error! Reference source not found., top-right) (Abson et al., 2013). A negative association with a higher SNGL share is expected to be due to reduced forage productivity and the limited market value of extensive beef cow systems (Bullock et al., 2011; Nishizawa et al., 2024). In contrast, areas with a higher suitability for arable production are expected to have higher farm gross margins (Fig. 2, top-middle).

Second, we hypothesise that landscapes with a higher yield potential are negatively correlated with the current biodiversity status (Fig. 2, middle-middle), while biodiversity is positively correlated with SNGL share (Fig. 2, middle-left) and landscape complexity (Fig. 2, middle-right) (Estrada-Carmona et al., 2022). This assumption can be supported by studies demonstrating that varied, less-intensively managed environments benefit species richness and habitat quality (Jonason et al., 2017; Öckinger and Smith, 2006).

Third, relative biodiversity–economic performance, by its definition in this study, is impacted by profitability and biodiversity. We expect that although high SNGL shares and higher structural landscapes provide lower profitability, their greater ecological benefits per unit economic performance will result in higher relative biodiversity–economic performance values (Error! Reference source not found., bottom-left and right). In contrast, we assume that it will be negatively correlated to yield potential (higher yield potential, lower relative biodiversity-economic performance) (Error! Reference source not found., bottom-middle).

### Bio-Economic Farm Modelling

To study the relationships that occur in each landscape window, we made use of a bioeconomic modelling approach developed by Nishizawa et al. (2024). This approach simulates the economically optimised production pattern of the entire county under various conditions that might influence the regional land use. To do this, the model maximises the total regional gross margin (Eq. 2), considering various factors that influence the farm-level gross margin from farming activities, including livestock, agricultural policies, available resources and input and output prices. Hence, the regional land use (production pattern) simulated by this model is understood as the manifestation of the aggregated result of land-use decisions under an individual farm. We assume that each of them engages in profit-maximising behaviour, which implies for policy-making that higher opportunity costs reduce the likelihood of adopting conservation measures unless adequately compensated.

The optimisation algorithm follows the general form of linear programming (LP) for n activities and m structural restrictions:

2 $$Z=\,{\sum }_{i=1}^{n}{c}_{i}* {x}_{i}$$

3 $${\sum }_{i=1}^{n}{a}_{{ij}}* {x}_{i}\le \,{b}_{j}\,{{\rm{for}}}\; {{\rm{all}}}\; {{\rm{j}}}=1,2,\ldots ,{{\rm{m\; and}}}$$

4 $${x}_{i}\,\ge 0{{\rm{for}}}\; {{\rm{all}}}\; {{\rm{i}}}=1,2,\ldots {{\rm{n}}}$$

Z is the total regional gross margin, x represents the decision variables for the farm activities, c denotes the contribution margin or variable cost per unit of activity, a is the technical coefficients and b represents resource availability (land and labour) or the upper/lower limits of activities.

Fixed costs, such as investments, paid labour, or rented land, were excluded from the optimisation, as the focus was on short-term decision-making in response to changing economic conditions. Therefore, all the costs considered in LP are directly related to a production level (i.e. variable costs). The LP operation was conducted with mathematical programming software (General Algebraic Modelling System—GAMS, version 31.2.0).

This original model accounted for the whole agricultural activity in Lääne County and three types of agricultural land are incorporated in this model: SNGL, permanent grassland and arable land. Various agricultural activities that are possible or conducted in the region are modelled for each land type, along with management intensities. Then the model determines the optimal cropping pattern in percentage shares for each land type as well as livestock numbers (i.e. number of dairy cows and beef cows) that maximise the aggregated gross margin of agricultural activities in the region. The fixed use of land types is assumed: the model prevents any expansion or reduction of the existing levels of each land type (i.e. SNGL, permanent grassland and arable land). The full list of modelled agricultural activities is provided in Supplementary Material 2. Building on this regional-level bio-economic farm model, our goal was to simulate the production pattern within the selected landscape windows. Then all three landscape factors that were explained in the previous section were calculated per landscape. To this end, the original model was adjusted as outlined in the following sections.

#### Semi-Natural Habitats

For this study, we used a GIS-based map to determine the area percentage of different habitat types in each landscape window (Table 3). The inclusion of different semi-natural habitat types allowed a more accurate gross margin calculation, as the biomass potential for feeding livestock, as well as compensation payments, varies across habitat types. Table 3 presents the newly obtained values of parameters to model these semi-natural habitat types.

**Table 3:** List of considered semi-natural habitat types in this study, the corresponding share of the total semi-natural grassland (SNGL) area, modelled management options and modelled parameters

Code^a^	Observed share (total 100%)	Modelled management options	Yield (t/ha)	Direct payment(€/ha)	Compensation payment (€/ha)	Metabolis-able energy^b^ (MJ/kg)	Crude protein (g/kg)	Crude fibre (g/kg)
1630	21.47%	Grazing	2.6	160	150	9.0	114	239
6280	0.02%	Grazing	1.1	160	250	9.0	103	226
9070	3.95%	Mowing	1.9	160	185	8.9	133	216
6450	44.09%	Grazing	6.3	160	150	8.9	117	227
		Mowing	7.4	160	85	8.9	117	227
7230	29.52%	Grazing	1.7	160	150	8.9	117	227
6510	0.49%	Mowing	5.4	160	85	8.9	117	227
6270	0.46%	Mowing	3.1	160	85	8.9	117	227

Direct payments are granted to land managers who manage their land in compliance with EU agri-environmental schemes, whereas compensation payments are granted specifically to land managers managing SNGL

^a^EU habitat directive. 1630 Boreal Baltic coastal meadows, 6280 Nordic alvar and Precambrian calcareous flatrocks, 9070 Fennoscandian wooded pastures, 6450 Northern boreal alluvial meadows, 7230 Alkaline fens, 6510 Lowland hay meadows, 6270 Fennoscandian lowland species-rich dry to mesic grasslands.

^b^Original metabolizable energy values are shown. In the modelling framework, values were multiplied by 0.6 to approximate grazing conditions and reduced effective forage utilisation under field conditions

While some landscape windows are covered by several types of habitats (landscape windows 1, 2 and 8, see Table 2), in others, only one habitat type is dominant (landscape windows 10, 11 and 12, see Table 2). As we obtained the area size of each habitat type, we calculated the area share and incorporated it into the model as an upper limit constraint. The typical management options of SNGL (either grazing, mowing, or mulching) were assigned to these habitats based on expert knowledge.

For both grazing and mowing options, synthetic input use was not allowed and in the case of mowing, we assumed only one cut per year. Biomass yields were gathered from several sources (Heinsoo et al., 2010; Melts et al., 2014; Melts and Heinsoo, 2015; Villoslada et al., 2018) and national grassland management studies (Estonian Environmental Board, 2021), as there was no single data source available that covered all the included habitat types. According to experts, for grazing, a 15% yield reduction compared to mowing was assumed.

Compensation payments for managing SNGL (Table 3) were taken from the Public of Estonia Environmental Board (2021). The payments are differentiated across habitat types as well as management options. Other parameters associated with habitat maintenance, such as cost, labour requirement and price of hay, were assumed to be identical across habitat types and the same values as in the original model were used.

The activities modelled for permanent grassland and arable land in this study are identical to those in the original model.

#### Farming Parameters Adjusted for the Landscape Windows

Table 4 presents the parameters that were used in the original model but adjusted for the scope of this study. As the original parameters were initially identified at a regional level, we needed to determine appropriate parameter values for each landscape window.

**Table 4:** List of modelled parameters that were adjusted from Nishizawa et al. (2024) for this study

	Landscape Window											
Adjusted parameters	1	2	3	4	5	6	7	8	9	10	11	12
Dairy cow capacity (LU)	0.0	0.0	3.5	0.0	5.1	2.9	2.1	0.0	0.5	1.6	0.0	0.0
Beef cow capacity (LU)	15.8	12.5	0.0	0.0	0.0	0.0	0.0	23.4	0.0	3.7	68.1	65.1
Hay export limit (t)	22.4	27.4	6.9	0.0	10.0	5.7	4.1	24.9	0.9	8.0	28.4	27.2
Biomass heating limit (t)	7.3	6.3	0.0	0.0	0.0	0.0	0.0	7.7	0.0	1.6	9.2	8.8
Labour availability (h)	518	421	345	227	376	301	275	608	246	344	1325	1267

Units *LU* livestock unit, *t* tonne, *h* hours

First, the livestock capacity of dairy cows and beef cows expressed in livestock units (LU) was identified as follows. This sets a limit for the maximal number of animals kept in each landscape window. For dairy cows, the maximum capacity was only considered for landscape windows that include arable land, as we assumed that concentrates produced from arable land are needed for this type of cow. Then, the maximum LU of dairy cows was calculated based on the area of permanent grassland and arable land at each site in a way that is proportional to the total number of dairy cows in the study region, considering the total area of permanent grassland and arable land. For beef cows, it was also determined by considering the area of SNGL on each site so that it is proportional to the total number of beef cows in LU and the area of SNGL in the study region. Additionally, as there is a large variability of biomass levels of the modelled semi-natural habitat types, we adjusted the maximum capacity for beef cows on each site, depending on the biomass levels of the habitat types, by using a weighted average of the biomass levels of all habitat types.

While the requirements for chemical feed components for dairy cows were maintained in the model as before, they were not considered for beef cows in this model, as SNGL is maintained primarily for biodiversity conservation purposes, supported by agri-environmental payments, rather than for productive beef production. This approach aligns with findings from Estonia, where semi-natural grasslands are often managed through low-intensity grazing or mulching, driven by compensation payments rather than livestock productivity considerations (Estonian Environmental Board, 2021; Villoslada Peciña et al., 2019).

The other parameters that were originally set at the regional level were distributed over the respective landscapes as follows. The total labour hours allocated for different site types (SNGL, permanent grassland, arable land) in the baseline scenario of the original model were downscaled to each landscape window based on the area of land use types, such that they can be proportional to the estimated labour requirements for each site type at the regional level. Additional labour requirements needed for managing livestock were added depending on the maximum capacity of dairy cows and beef cows.

The hay export demand at the regional level was proportionately distributed to each landscape window depending on the area of permanent grassland. Biomass demand for a heating plant at the regional level was distributed depending on the SNGL area. The observed area of SNGL, permanent grassland and arable land in the GIS data was used as the upper limit of land use at each site. As is in the original model, it remains impossible to expand the given area.

### Assessing Farmland Biodiversity

To indicate the habitat quality for regionally relevant bird species as a proxy for farmland biodiversity, we utilised habitat scores calculated with the habitat value model (HVM) (Brandt and Glemnitz, 2014; Glemnitz et al., 2015). The HVM model determines the habitat quality of agricultural land for indicator bird species (Brandt and Glemnitz, 2014).

To this purpose, it first assesses the habitat preferences of indicator bird species per species by considering breeding period, preferences for the crop coverage for breeding and feeding habitat and preferences for the crop height for breeding and feeding habitat according to Brandt and Glemnitz (2014).

Then it assesses the potential habitat suitability of all agricultural land use activities by their provided vegetation structure, their cultivation period of crops in 10-day periods and necessary agricultural measures (Brandt and Glemnitz, 2014). The breeding habitat suitability definition by Brandt and Glemnitz 2014 was used, i.e. at least two consecutive 10-day periods fulfil the requirements of the bird species and during this time and an additional 10-day period, any agricultural measures were not allowed (e.g. grass cutting, harvesting). The feeding habitat suitability definition by Brandt and Glemnitz (2014) encompassed a 10-day period, which fulfils the requirements of the bird species. The number of available feeding periods of each agricultural activity was assessed.

In a third step, the habitat preferences of each considered bird species and the potential habitat suitability were overlaid. Two indices per indicator species were derived – a breeding habitat index, expressing the relative number of successful breeds per species and year and a feeding habitat index, expressing suitable habitat conditions for bird feeding. The index values for single species were aggregated to allow for a better interpretation.

In the last step of HVM modelling, a multiplication of habitat values with the area percentage of each activity allows for an aggregation of results to the regional scale (here, landscape window) for each model run (Glemnitz et al., 2015).

The species selection of the HVM model refers to the farmland bird indicator (BMU, 2007) and was adjusted to relevant species of the Estonian case study region by local bird experts (Nishizawa et al. 2024). They were chosen as being typical for agricultural landscapes, sensitive to management changes and covering a variety of different habitat requirements. For this study, we further differentiated the habitat scores for arable and grassland to better reflect differences in habitat quality of each land cover type. To this end, we decided to use the species corncrake (*Crex crex*) and grey partridge (*Perdix perdix*) to represent the potential habitat quality of grassland by these indicator species. The corncrake prefers to live in the grassland if available. The partridge has good habitat conditions in extensive grassland. Both are mainly relevant to represent grassland habitats of the grassland-rich case study region and were thus used as indicators for them. We used Eurasian skylark (*Alauda arvensis*), northern lapwing (*Vanellus vanellus*), whinchat (*Saxicola rubetra*), red-backed shrike (*Lanius collurio*) and yellowhammer (*Emberiza citrinella*) as indicator species for the arable land to represent the habitat quality of arable land in this case study region. This does not mean that the selected species cannot also choose other habitats in the agricultural landscape; it only means that they are particularly suitable for regionally representing the habitat quality of the respective habitats.

From all index values, the average scores over species for feeding and breeding index were calculated and then they were normalised to a 0–1 scale to assess the aggregated suitability of each crop and grassland usage, resulting in four categories of habitat scores: grassland breeding index, arable land breeding index, grassland feeding index and arable land feeding index.

**Fig. 3:**
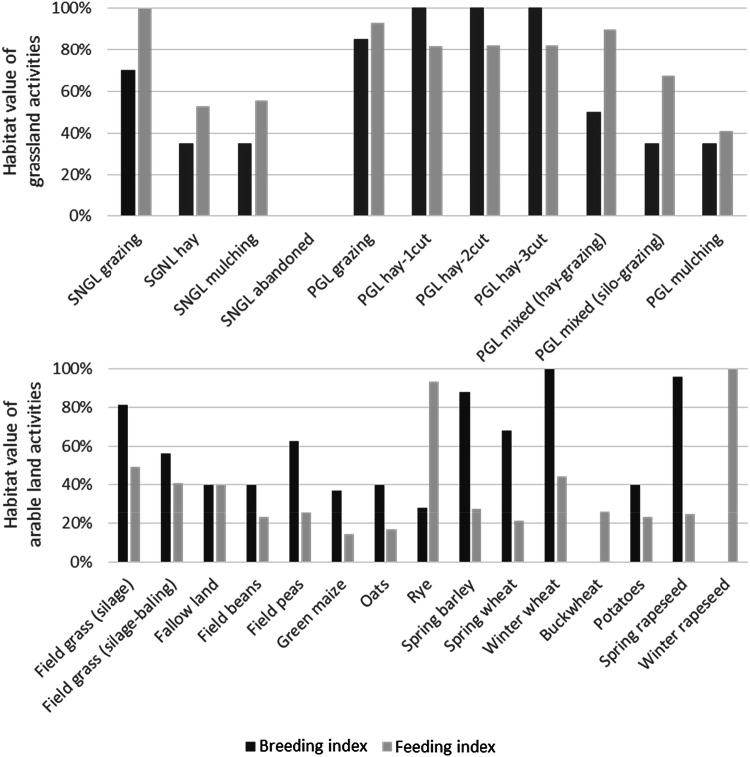
Normalised habitat values of grassland activities (above) and arable crop activities (below) for the feeding and breeding bird index calculated with the HVM. SNGL semi-natural grassland, PGL permanent grassland. Due to the normalisation of values, a comparison of values across different activities can be made only among grassland or crop activities

Figure 3 shows the resulting habitat values of grassland (top) and arable activities (bottom) from the HVM. Due to the normalisation of values, a comparison of values across different activities can only be made within grassland or crop activities.

There are some differences in the values between SNGL and permanent grassland activities in the feeding index: the feeding index was mostly higher on permanent grassland. Many bird species benefit from the low vegetation structure after mowing/cutting on permanent grassland, as this makes it easier for them to access insects on the ground surface. However, both indicator species have, according to local bird experts, opposite requirements for the feeding habitat quality. The Grey partridge searches for fodder in more open land with a comparable low vegetation height of 10–30 cm and thus benefits from more than one cut. The Corn crake needs protection by vegetation, also in the search for fodder with a comparable high vegetation height of 30–60cm and thus doesn’t benefit from more than one cut. These opposing trends for the two included bird species counterbalance each other mathematically.

The differences in the breeding index for the values between SNGL and permanent grassland activities show that the permanent grassland with cutting is higher than the other grassland activities and similar in the three cuts. The reason is that the index results for breeding indices of two birds: The corn crake and the Grey partridge. Both birds are typical Grassland-Breeders and require a medium vegetation density as shelter for their nests against predators. Grey partridge and Corncrake benefit both from the habitat quality for breeding in the grassland activities PGL hay cut 1, for PGL hay cut 2 and PGL hay cut 3; the other grassland activities result often in a lower grassland density and/or more disturbances. Thus, PGL hay cut 1, PGL hay cut 2 and PGL hay cut 3 achieve a breeding index of ten (1 suitable breed). The one breed can be achieved before cuts 1, 2 and 3.

The values of crop activities varied widely both among activities and land cover types. Field grass, spring barley, spring wheat, winter wheat and spring rapeseed provide higher feeding habitat values, while rye and winter rapeseed provide higher breeding habitat values.

### Forward-Looking Scenario Analysis of Biodiversity Enhancement

In addition to assessing the baseline relative biodiversity–economic performance of biodiversity provision under economically optimised land-use patterns simulated based on the current status, we conducted a hypothetical scenario analysis to explore the economic implications of increasing biodiversity outcomes across different landscape windows. This analysis does not represent current farming practices or observed policy outcomes, but rather serves as an exploratory tool to assess the relative economic constraints associated with enhancing habitat values in contrasting landscape contexts.

Specifically, for each landscape window, we imposed an exogenous increase in habitat value scores relative to the baseline outcome and quantified the resulting income loss per landscape window. By comparing the magnitude of income loss across landscape windows, this approach allows us to assess where additional biodiversity measures could, in principle, be implemented at lower economic cost. The results of this analysis are therefore intended to inform strategic targeting of future conservation efforts, rather than to evaluate the relative biodiversity–economic performance of existing policy instruments.

### Using Remote Sensing Data for Validating the Modelled Yield

To validate the assumption of constant yield levels across landscape windows, we additionally tested for a subset of landscape windows (8–12) how the use of remote sensing data would influence the modelling results. To this end, we used normalised difference vegetation index (NDVI) values from Venter et al. (2024). The NDVI is widely used and was employed in this study as a relative yield index to adjust the previously collected grassland yield levels (Bretas et al., 2021; Guerini Filho et al., 2020). NDVI was calculated using Sentinel-2 satellite imagery from 2021, with cloud masking applied using Cloudscore Plus (Venter et al., 2024). The remaining cloud-free images were composited by taking the median spectral value per band over the year. NDVI values were then linearly normalised between 0 and 100, using the 5th and 95th percentiles of raw NDVI values as the minimum and maximum for normalisation. Thus, a value of 100 corresponds to productivity above the 95th percentile, while a value of 0 corresponds to productivity below the 5th percentile.

The NDVI yield index values were aggregated into an area-weighted average per land use category (SNGL, permanent grassland, arable land). To apply these values, we assumed that the previously modelled yield levels, based on available data excluding satellite observations, represented the average productivity, corresponding to a value of 50 on the NDVI yield index scale. The NDVI yield index was then used to adjust the original yield levels for each landscape window. Specifically, the original yield was scaled proportionally to the NDVI yield index for the corresponding land use category in that landscape window. Table 5 presents the resulting area-weighted NDVI index for SNGL, permanent grassland and arable land. The final factor used to adjust the original yield levels of agricultural activities was calculated according to Eq. (5):

5 $${Adjusted}\,{yield}={original}\,{yield}\,{level}* ({area}\,{weighted}\,{NDVI}\,{index}/50)$$

**Table 5:** Area-weighted NDVI index for grass yields in the landscape windows from 8 to 12. The index is relative to the average productivity, corresponding to a value of 50

	Landscape window with remote sensing data				
Area-weighted NDVI index	8	9	10	11	12
SNGL	52.3	–	80.5	70.3	80.3
Permanent grassland	85.9	46.4	72.8	–	–
Arable land	–	39.3	63.0	–	–

For example, the pre-existing yield level for grazing of Boreal Baltic coastal meadows (code: 1630) in landscape window 8 is 2.6 t/ha and the area-weighted NDVI yield index for SNGL in the same site was 52.3; the adjusted yield was calculated as 2.6*(52.3/50) = 2.7 t/ha. This adjustment was applied separately for each landscape window (8–12) to refine the spatial variability of modelled yield levels based on remote sensing data. The NDVI index for arable land was applied to the yields of field grass on arable land.

## Results

### Cropping Pattern and Landscape Complexity

**Fig. 4:**
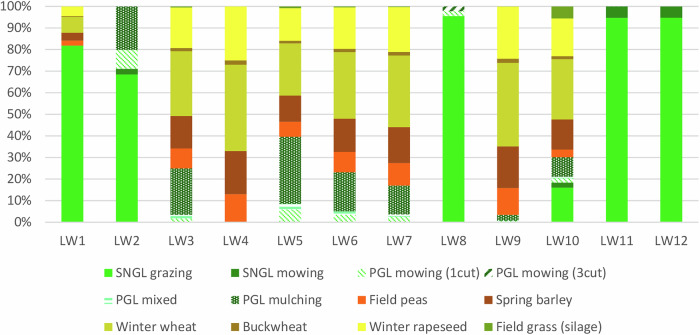
Economically optimised cropping pattern (percentages) of the 12 landscape windows (LW); SNGL semi-natural grassland, PGL permanent grassland

Figure 4 shows the economically optimised farm activities on SNGL, permanent grassland and arable land across the respective landscape windows as simulated by the model. Most of the SNGL is used for grazing due to its higher compensation payment, while a large part of the permanent grassland is only mulched ( = minimum requirement for compensation payments). The chosen arable crops included winter wheat, rapeseed, spring barley and field beans, which together account for 90% of the arable land. Table 6 presents the resulting Shannon diversity index calculated based on the simulated cropping pattern on arable land and the final landscape complexity index used for the analysis. As the combination of arable crops and the shares within arable land showed a similar pattern, there is only a slight difference in the Shannon diversity index across the landscape windows. The final landscape complexity index, however, varies across the landscape windows. The landscape window with the highest value is LW (landscape window) 8, followed by LW1, LW2, LW11 and LW12. All the landscape windows are predominated by SNGL and harbour a fair amount of landscape structural elements. Overall, a higher grassland share, including permanent grassland, leads to a higher landscape complexity index.

**Table 6:** Resulting Shannon diversity index and the final landscape complexity index across the landscape windows

	Landscape windows											
Composite indicator	1	2	3	4	5	6	7	8	9	10	11	12
SNGL share	0.82	0.71	0.00	0.00	0.00	0.00	0.00	0.96	0.00	0.18	1.00	1.00
Landscape structure index	0.56	0.76	0.30	0.23	0.87	1.00	0.23	0.91	0.45	0.71	0.45	0.58
Crop Shannon diversity index	0.69	NA	0.70	0.69	0.71	0.70	0.70	NA	0.69	0.72	NA	NA
**Landscape complexity index**	**0.69**	**0.74**	**0.33**	**0.30**	**0.53**	**0.57**	**0.31**	**0.93**	**0.38**	**0.54**	**0.72**	**0.79**

The values of SNGL share and landscape structure index were predetermined before the model-run

### Profitability

**Fig. 5:**
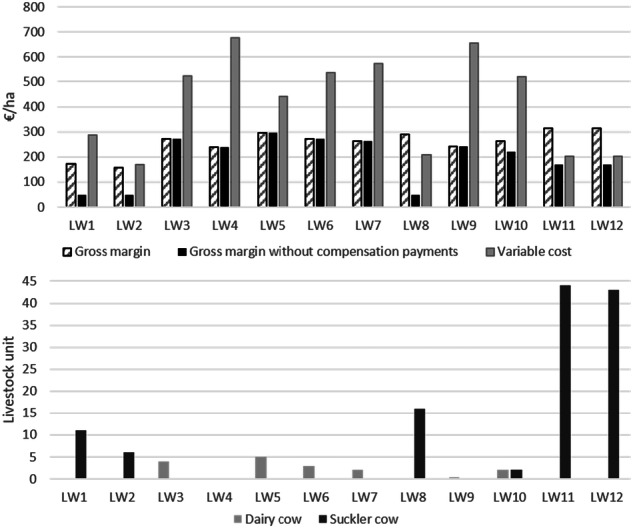
(top) Resulting gross margin per hectare, gross margin without compensation payments for the activities on SNGL and variable cost per hectare across the landscape windows (LW), based on the economically optimised cropping pattern. (bottom) Total livestock numbers in livestock units across the landscape windows

Figure 5 (top) shows three economic indicators that resulted from the simulated cropping pattern shown in Fig. 4: gross margin per hectare, gross margin per hectare without compensation payments for the activities on SNGL and variable costs per hectare across the landscape windows. Figure 5 (bottom) presents the optimised number of livestock animals in livestock units across the landscape windows.

The most profitable landscape windows, including compensation payments, were LW8, LW11 and LW12. These landscape windows are mostly covered by SNGL and the higher profitability on these sites is secured with compensation payments. Without compensation payments, their gross margin would be lower compared to landscape windows with arable land. Particularly, LW8 resulted in the lowest gross margin due to fewer SNGL and smaller livestock numbers. Considering the profitability without compensation payments, the landscape windows that are predominantly covered by arable land (LW3, LW4, LW6 and LW7) had the highest profitability despite higher variable costs. Figure 5 (bottom) shows that the livestock numbers of suckler cows are particularly high in LW11 and LW12 because of the higher yield levels of the SNGL habitats in these LWs.

**Fig. 6:**
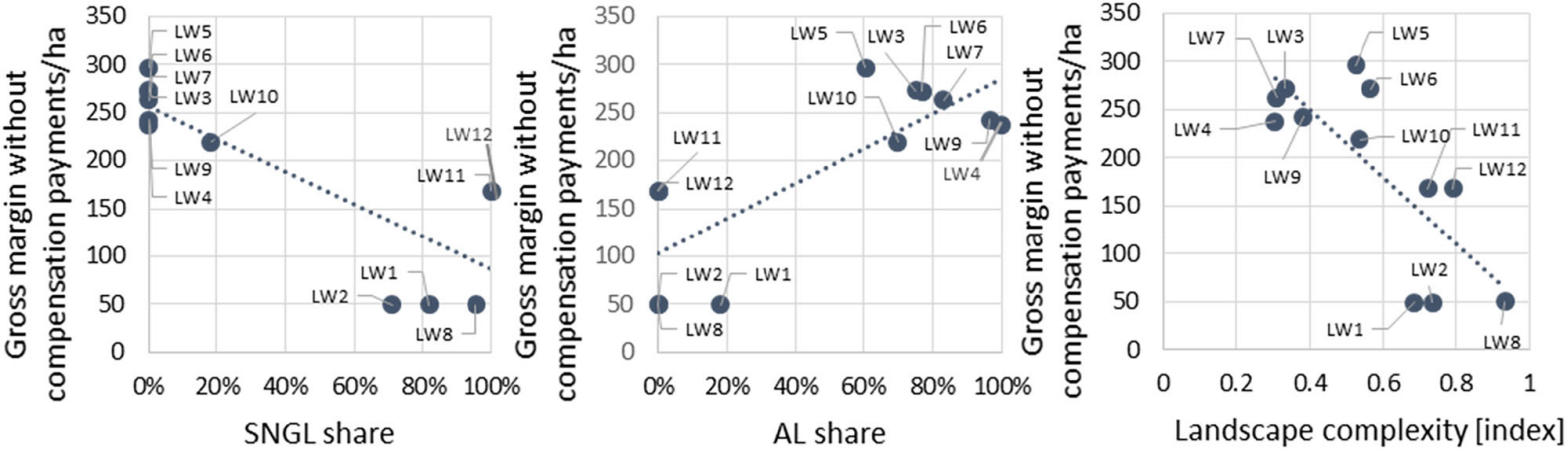
Scatter plots of the resulting landscape windows (LW) to indicate how the share of SNGL, arable land share and landscape complexity affect the profitability of farm activities within each landscape window. Biodiversity

Figure 6 illustrates the relationship between the indicators representing varied landscape factors and farm profitability, given the simulated cropping pattern presented in Fig. 4. Please note that the lines in the following scatter plots suggest a tendency between the factors and the outcomes (i.e. not a statistically represented regression). The analysis reveals that all the results support the hypotheses: landscape windows with a higher SNGL share and landscape complexity resulted in lower gross margin, while landscape windows with a higher arable land share resulted in higher gross margin. As explained in Fig. 5, compensation payments for the activities on the SNGL mask the higher profitability of landscape windows with a higher SNGL share. However, their gross margin without compensation payments is lower than that of the other landscape windows, while the gross margin of arable land is particularly high. Furthermore, landscape windows with a higher SNGL share (LW1, LW2, LW8, LW11 and LW12) tend to have a more complex landscape structure. Therefore, a similar trend between SNGL shares and gross margin is also observed for landscape complexity and gross margin.

### Biodiversity Values

**Fig. 7:**
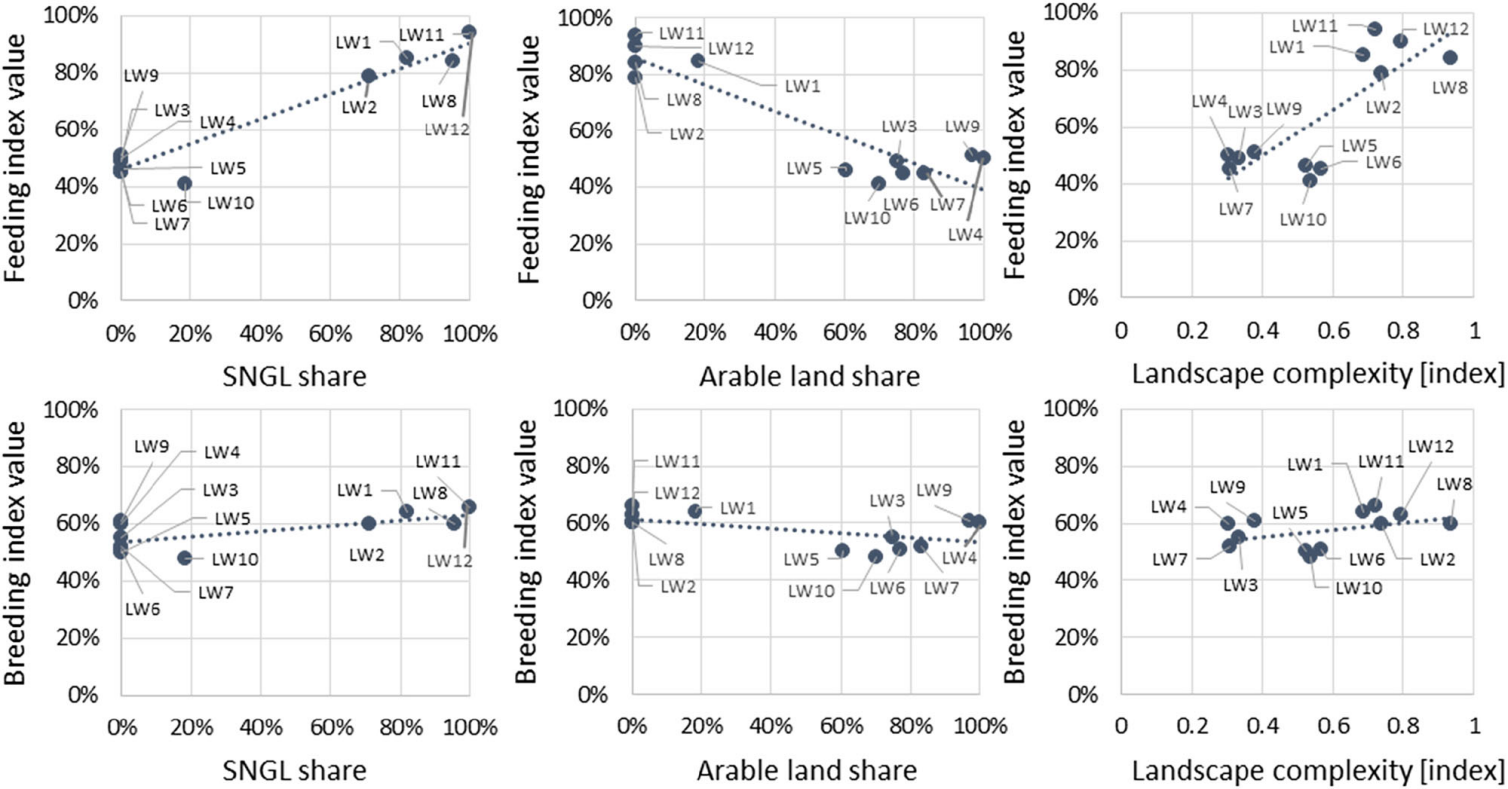
Scatter plots of the resulting landscape windows (LW) to show how the share of SNGL, arable land share and landscape complexity affect the profitability of farm activities within each landscape window

Figure 7 shows the trends between habitat values of birds for feeding and breeding and the single tested landscape factors. Habitat values tend to increase with greater SNGL share and landscape complexity, while they decrease with higher arable land share. These trends are more pronounced for feeding habitat values than for breeding. This difference likely stems from the fact that the habitat value for breeding is more driven by distinct qualitative parameters and related to a limited number of bird species, as part of the list of selected bird species. Moreover, for breeding, most of the birds occupy territories and keep a distance from the next pair of breeding birds. As a result, improvements in the heterogeneity of different habitats or increases in the share of positive elements in landscapes result only in limited increases in the breeding bird values. For the feeding index, increasing the area of SNGL or cropping diversity and landscape structure leads to stronger additive effects for nearly all species from the defined indicator species list. Increases in landscape complexity, therefore, count stronger for feeding habitat use than for feeding use. Moreover, there is a larger gap between the habitat values of the chosen grassland activities and arable crop activities for feeding birds. Landscape complexity positively contributes to the habitat values, with a similar reason for profitability: the landscape windows with a higher SNGL share tend to have a higher landscape structure.

### Relative Biodiversity–Economic Performance

**Fig. 8:**
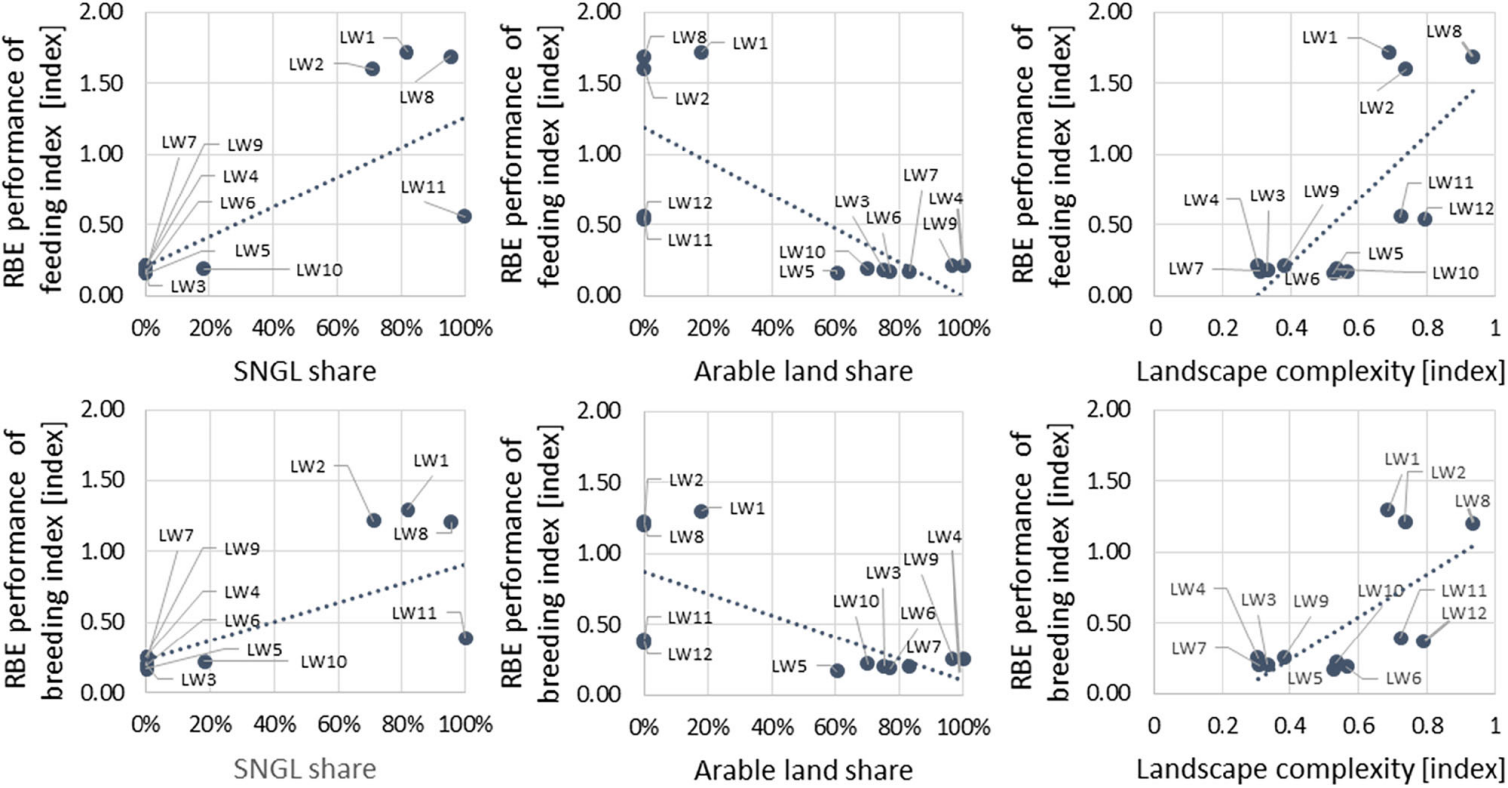
Scatter plots of the resulting landscape windows (LW) to show how the share of SNGL, arable land share and landscape complexity affect the relative biodiversity–economic performance of farm activities within each landscape window. RBE relative biodiversity-economic (performance)

Figure 8 reveals that relative biodiversity–economic performance for both feeding and breeding bird habitat use is higher in landscapes with a higher share of SNGL and a higher level of landscape complexity. This outcome supports the hypothesis that SNGL and landscape complexity positively affect relative biodiversity–economic performance. Regarding yield potential, the hypothesis was that yield potential has a negative impact on relative biodiversity–economic performance. In contrast, the result shows that in a landscape with a higher share of arable land, relative biodiversity–economic performance is lower. This outcome can be explained by a relatively higher gross margin and lower habitat values of the landscape windows with a higher share of arable land. The same is true for the outcomes for landscapes with SNGL shares and landscape complexity: the landscape windows with a higher SNGL share and a higher landscape complexity resulted in higher habitat values while obtaining a lower gross margin.

### Trade-offs between Gross Margin and Habitat Value

**Fig. 9:**
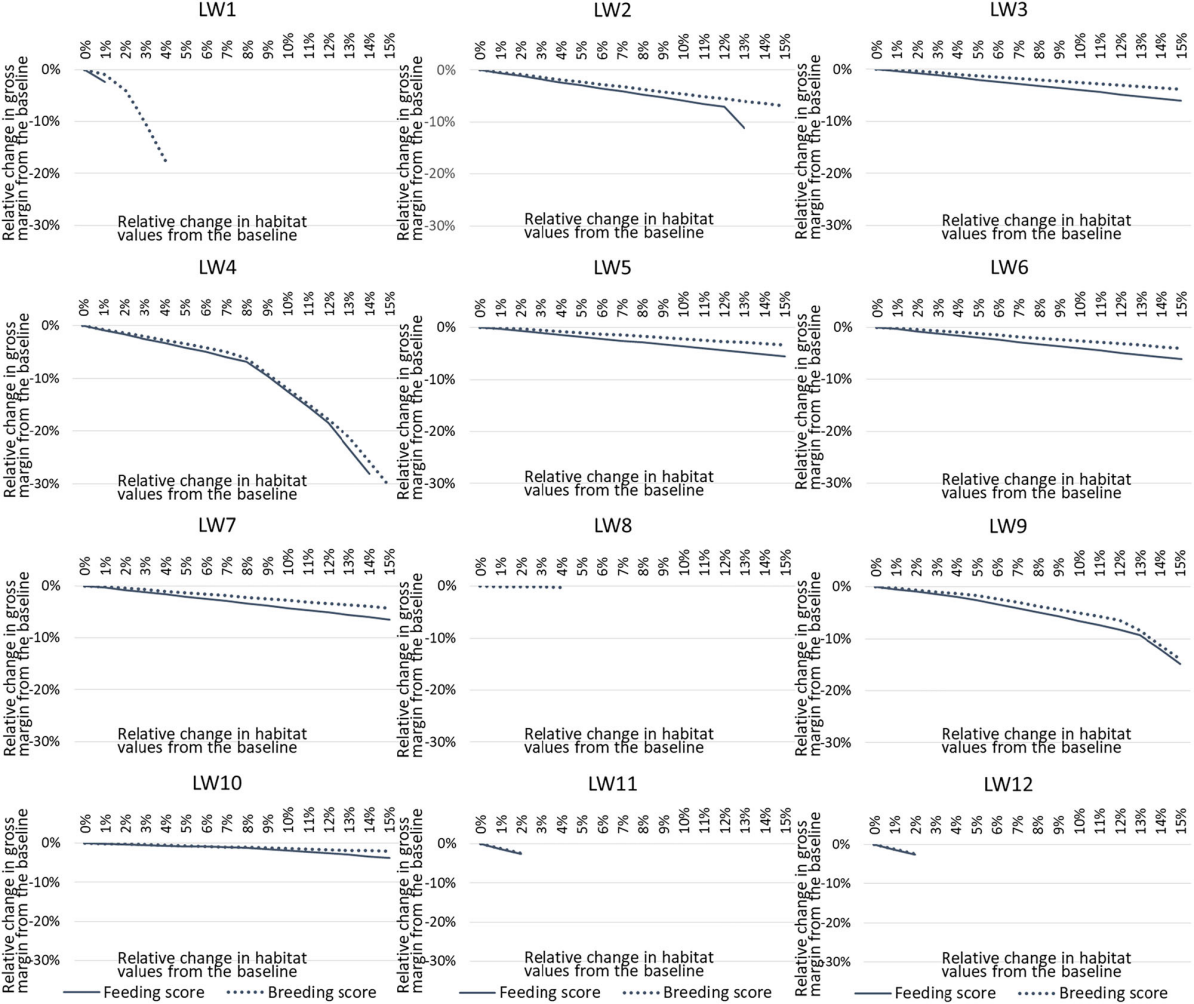
(Y-axis) Relative change in gross margin from the baseline across the landscape windows (LW) when compelling the model to enhance the habitat values for feeding and breeding from the baseline by one percent to a maximum of 15% (X-axis)

Figure 9 shows the resulting trade-offs between gross margin and the aggregated habitat value for each landscape window, obtained from a forced stepwise increase in aggregated habitat value compared to the baseline scenario. The steeper the slope of the curves, the greater is the decrease in gross margin relative to habitat values. The maximum increase in habitat values (up to 15%) reflects the room for feasible model solutions. Beyond these values, the model could not find a feasible solution to increase the values further.

The landscape windows that are dominated by SNGL (LW1, LW8, LW11, LW12) had fewer possibilities to further improve the habitat values compared to the reference scenario, as most SNGL is used for grazing in the reference scenario and there was no alternative activity for SNGL that provides better habitat values than grazing. Among the other landscape windows, LW3, LW5, LW6, LW7 and LW10 showed a relatively mild income loss as habitat values increase, compared to the income loss at landscape windows LW4 and LW9. These LWs have in common some area of permanent grassland, while LW4 and LW9 are almost entirely covered by arable land.

### Validation of the Modelled Yields with Remote Sensing Data

**Fig. 10:**
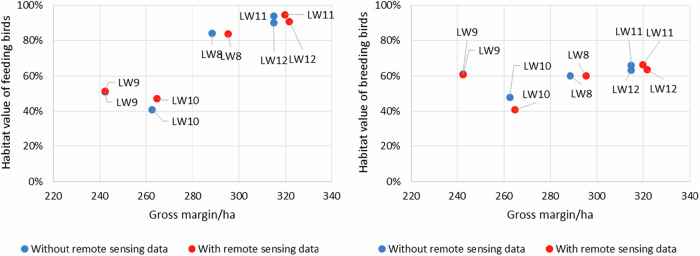
Adjusted gross margin and habitat values of feeding birds (left) and breeding birds (right) for a subset of the landscape windows (LW8, LW9, LW10, LW11, LW12) by replacing the original yield levels with the refined yield levels with the remote sensing data

In Fig. 10, the change in gross margin and habitat values for feeding and breeding birds is displayed after adjusting the original yield levels used for calculating the baseline results with remote sensing data.

As shown, the inclusion of differentiated yields from remote sensing slightly changed the position of the respective landscape windows in the graph, yet led to no change in the overall trend, emphasising the robustness of the above-presented results. Nonetheless, their inclusion allows for an improved gross margin calculation and thus also a more accurate relative biodiversity–economic performance calculation.

## Discussion

### Landscape Factors and Profitability

Our analysis showed that landscape windows with a higher share of SNGL have lower profitability when compensation payments for SNGL are not considered, which is consistent with the initial hypothesis supported by Abson et al. (2013). The beef cows that graze grass on SNGL in the summer and are fed roughage in the winter fatten slowly due to low yields and low nutritional values (Kalle et al., 2025). Hence, such pasture-based beef production is generally less profitable (Holmström et al., 2021). In reality, most farmers maintain SNGL not to make profits through beef production but to receive compensation payments (Kalle et al., 2025), which in turn brings benefits for nature conservation (e.g. preventing wood encroachment and preserving unique species developed on the sites). An exception is found at the sites with higher grass yields and more beef cows, such as LW11 and LW12, which can still be profitable. This finding indicates that fully utilising livestock capacity, considering yield levels, could be a key factor in achieving economic sustainability for raising beef cows on SNGL. In contrast, the landscape windows dominated by arable land had higher profitability, despite their high variable costs associated with arable farming: cultivating arable crops generates much higher revenues for farmers, which can easily compensate for their high production costs, as evidenced by ongoing land concentration in rural areas in Estonia (Rasva and Jürgenson, 2020). Our hypothesis for a negative relationship between landscape complexity and profitability was also confirmed, as supported by Latruffe and Piet (2014), who demonstrated a negative impact on farm performance due to land fragmentation. This is mainly because those considered landscape elements (trees, tree rows and stone walls/heaps) are more present on grass sites than on arable land.

### Landscape Factors and Biodiversity

Regarding biodiversity, the results confirm that while a higher SNGL share and more landscape structural elements positively influence habitat values, which is also confirmed by Estrada-Carmona et al. (2022), the arable land share was negatively correlated with habitat values, particularly for the feeding index. This contrasting trend can be explained by the fact that habitat values of grassland management were generally higher than those of arable crop activities, e.g. due to the lack of periodic soil tillage. The habitat values of arable crop activities showed a wide variation (Fig. 3), depending on the crop types and their management. Some activities provide high feeding habitat values, such as spring barley, winter wheat and spring rapeseed, while rye and winter rapeseed can serve as breeding habitats. This suggests that, depending on the spatial and temporal combination of crops, sites that are dominated even by arable land can still achieve high habitat values, which is in line with Haughton et al. (2016), who demonstrated that the strategic choice of arable crops could enhance biodiversity in landscapes dominated by arable land.

### Interpretation of Outcomes for Relative Biodiversity–Economic Performance

The results for the relative biodiversity–economic performance analysis were largely in line with our hypotheses. Landscape windows with higher SNGL shares not only had a higher habitat value but also demonstrated a relatively efficient use of financial resources.

Nevertheless, as presented in Fig. 9, enhancing habitat values beyond a certain threshold, particularly in SNGL-dominated sites, may result in larger economic losses, or further improvements may be extremely limited by inherent land use constraints. In contrast, arable land-dominated sites showed possibilities to improve habitat values with mild losses in profitability. This finding suggests a lower opportunity cost of choosing an alternative cropping pattern to enhance bird habitats on arable land (see also Sidemo-Holm et al., 2024).

### Methodological Reflections

#### Landscape Window Approach With Modelling

Kay et al. (2018) applied a similar landscape approach to assess the provision of different ecosystem services along a gradient of agroforestry and arable land use within selected landscapes, yet without economic parameters. By including an economic aspect in our approach, we made it possible to conduct a landscape window-specific analysis with different outcomes across economic and ecological dimensions. Compared to a regional aggregated study, this methodology potentially allows for creating artificial land use maps at a higher resolution, at which level changes in agricultural landscapes and their ecological outcomes occur (Langhammer et al., 2019). However, some bias may arise if the results of this study were generalised to the entire region, as the selected 12 landscape windows are not statistically representative of the landscape characteristics of the entire region. Although spatial autocorrelation among the windows was found to be non-significant, this does not imply representativeness at the regional scale. Furthermore, while the selected threshold of the agricultural land within landscape windows ensured compatibility with both economic-ecological modelling frameworks, alternative thresholds could also be explored in future research.

Furthermore, our outcomes on profitability and biodiversity are not dependent on a spatial allocation of crop activities over landscape windows. i.e. individual fields and their location are not considered in the analysis. Several studies demonstrated that smaller fields were associated with multiple benefits to farmland biodiversity (Concepción et al., 2020; Sirami et al., 2019). Although detailed crop information exists in Estonia’s IACS/LPIS systems and is increasingly made available through harmonised datasets such as EuroCrops (Jänicke et al., 2025), these observed crop data were not included in this study due to limitations in temporal coverage and the complexity of harmonising multi-year field data for the landscape windows used here. Despite this limitation, this study provides some insights into economic and biodiversity interaction in heterogeneous landscapes. Future research can incorporate a statistical method for selecting landscape windows to be able to extrapolate to other regions and refine the assessment of biodiversity with the potentially varied effects of spatial distributions of fields and landscape structures.

Last, studies on estimating grassland productivity using remote sensing data have only recently emerged in Europe (Bangira et al., 2023). Therefore, the scarcity of validation data is a challenging obstacle in grassland remote sensing and a high sensitivity of the applied models to the resulting potential estimation of grassland yields exists (Lange et al., 2022; Schwieder et al., 2022). This study was also restricted by the limited availability of the remote sensing data. They were only applied to half of the selected landscape windows and used exclusively for validation purposes rather than as the main inputs to the modelling framework. To strengthen the validation procedure and guarantee the accuracy of the estimated grassland productivity, researchers must collect more ground-truth grassland data in Europe. Cooperation between scientists, land managers and legislators is crucial, as grassland management data are often recorded only on the field level (Lange et al., 2022).

#### Indicator Choice For Representing Landscape Complexity

Our composite indicator that represented landscape complexity included the Shannon diversity index, which is the most frequently used metric to represent landscape heterogeneity (Tonetti et al., 2023). Nonetheless, including more aspects of heterogeneity can be useful to improve the assessment of biodiversity (Penko Seidl and Golobič, 2020; Redon et al., 2014). For example, Lu et al. (2024) identified a large variation in bird richness depending on the choice of metrics, such as structural (land use pattern) or functional (landscape features) landscape complexity, landscape composition and landscape heterogeneity. This study created an indicator without providing a strict definition for landscape complexity from a biodiversity point of view, which remains an issue for further studies. Also, land use intensity should be more explicitly considered for farmland biodiversity assessment, as landscape simplification is not necessarily an indicator for agricultural intensification (Persson et al., 2010; Roilo et al., 2024).

### Policy Recommendations

Several policy implications can be made given the resulting tendencies and patterns between economically optimised cropping patterns, profitability and biodiversity outcomes within heterogeneous landscapes. We acknowledge that farmers’ responses to agri-environmental measures are also shaped by non-economic values (e.g. social norms, access to knowledge and transaction and administrative costs, etc.) and biodiversity value remains the primary justification for conserving semi-natural grasslands. However, the policy implications discussed here have been framed solely under the fundamental assumption of the employed model that farm managers are to maximise economic performance (i.g, profits) over economic or non-economic values. To discuss the results at a policy level, we see our selected landscapes as owned by an individual farm.

First, continual financial support needs to be given to the farms that own a larger share of SNGL. There should be a rise in the payments for managing semi-natural habitats that produce lower biomass, as these sites hinder the revenue creation from beef production. Current payments for maintaining SNGL are differentiated primarily based on their management methods and the presence of trees, but not based on potential biomass yield in Estonia. More accurate knowledge of the yields of semi-natural habitats can facilitate a more balanced payment system for economic and ecological goals. Such a strategy can lead to the preservation of the already existing landscape elements, which are often seen on SNGL.

Second, even though the biodiversity level is lower on farms with a higher share of arable land, a more elaborate combination of arable crops can lead to better farmland biodiversity. Currently, direct payments to arable crops are uniform and a crop rotation scheme is mainly determined by its impacts on soil health and phytosanitary considerations. Therefore, adequate incentives for crop rotations that promote farmland biodiversity are recommended.

As landscape windows dominated by arable land exhibited potential for enhancing habitat values with a slight reduction in profitability, such policies can be justified from the point of economic performance. Although the design and effectiveness of grassland-focused conservation measures remain limited across many other EU countries (Staggenborg and Anthes, 2022), this study, which focused on a grassland-rich area in Estonia, demonstrated the relevance of considering the impacts of both grassland and arable land management on farmland biodiversity conservation. Policymakers should therefore adopt a more integrated landscape-level perspective that accounts for interactions among different land-use types beyond the impacts of individual farm activities and create more economically and ecologically effective measures (König et al., 2015).

## Conclusion

This study explored the interplay between profitability and biodiversity within heterogeneous landscapes for facilitating economically efficient grassland conservation and biodiversity measures in Estonia’s grassland-rich region. For this purpose, we developed an approach for a landscape-level assessment and incorporated it into an existing bio-economic farm model.

We found that higher landscape complexity was associated with higher levels of biodiversity, while profitability was lower. Particularly, an SNGL-dominated area with lower grass yields suffers the lowest profitability due to lower capacity for beef cows while providing higher biodiversity. In contrast, an area covered with more arable land than grassland can be targeted for biodiversity for the better relative biodiversity–economic performance of biodiversity measures. Therefore, conservation policy should protect high biodiversity landscapes from further declines in profitability and find suitable measures to enhance biodiversity in arable land-dominated areas without significantly affecting their profitability.

Future research should advance spatially explicit and representative landscape-level modelling by incorporating field-level land-use patterns, improved grassland productivity validation and landscape indicators that distinguish between composition, configuration and land-use intensity, considering their ecological processes. Such efforts are needed to achieve precise, differentiated, landscape-scale agri-environmental payment schemes that enhance economically efficient biodiversity conservation measures.

## Supplementary information

Supplementary information

Supplementary information

## Data Availability

No datasets were generated or analysed during the current study.
