# Supplementary material for: How landscape factors relate to biodiversity-economic performance in an Estonian grassland-rich region
Source: Environ Manage. 2026 Jul 7;76(7):246. doi: 10.1007/s00267-026-02533-x (PMC13342203; doi:10.1007/s00267-026-02533-x)
Supplement: Supplementary file 1 — Supplementary information [file 267_2026_2533_MOESM1_ESM.docx]

**Supplementary material 1: Method for selecting landscape windows and their location within the Lääne County**

**(Takamasa et al.: How landscape factors relate to biodiversity-economic performance in an Estonian grassland-rich region)**

Landscape windows were selected to represent contrasting agricultural landscapes within Lääne County. To this end, landscape windows were defined as 1 × 1 km squares determined by a centroid point. Using QGIS, we generated 60 random points within areas classified as agricultural land according to the Estonian basemap in Lääne County, where each point defined the centroid of a candidate landscape window.

For each window, land-cover composition was calculated based on the available geospatial datasets. Three criteria were applied to keep eligible windows: (I) the window is predominantly agricultural land; (II) the window is not crossed by main roads and contains hardly any settlement areas; and (III) the combined proportion of built-up areas, forest, and water is <20%, i.e. at least 80% of the area consists of agricultural land (arable land, permanent grassland, or semi-natural grassland). Windows not fulfilling these criteria were excluded.

To minimise potential spatial autocorrelation and avoid spatial overlap, a minimum distance of 2 km between window centroids was enforced during the selection process. From the remaining eligible windows, we characterised them according to the share of semi-natural grassland (SNGL), resulting in six windows without SNGL and six windows containing SNGL.

During the final selection step, three initially chosen windows were slightly repositioned to the nearest locations fulfilling all predefined criteria (agricultural land threshold, exclusion of roads and settlements, SNGL classification, and ≥2 km minimum distance). To ensure that we did not introduce spatial autocorrelation as a result of this process, we quantified spatial dependence among the final set of 12 landscape windows using Moran’s I. Window centroids were used to construct a k-nearest-neighbours spatial weights matrix, and significance was assessed using permutation tests implemented in the spdep package in R.

The resulting set of 12 landscape windows is spatially distributed across Lääne County (Figure 1) and forms the basis for the subsequent modelling analyses.


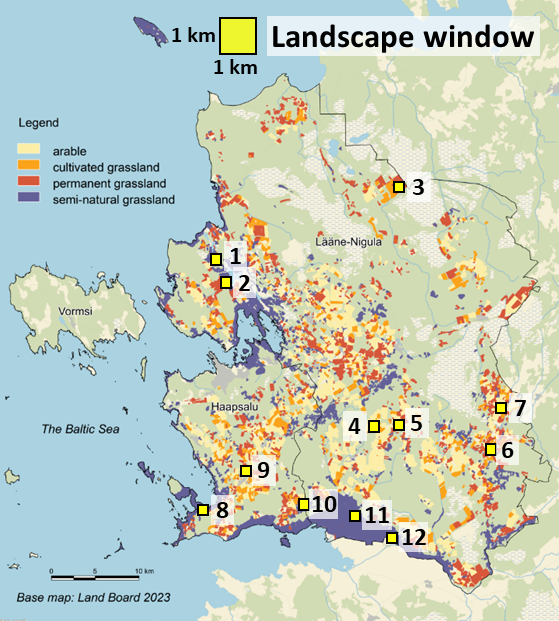


Figure 1: Location of the landscape windows and the map of agricultural land use categories in the case study region Lääne County.
