# Supplementary material for: How landscape factors relate to biodiversity-economic performance in an Estonian grassland-rich region
Source: Environ Manage. 2026 Jul 7;76(7):246. doi: 10.1007/s00267-026-02533-x (PMC13342203; doi:10.1007/s00267-026-02533-x)
Supplement: Supplementary file 2 — Supplementary information [file 267_2026_2533_MOESM2_ESM.docx]

**Supplementary material 2: List of the modelled farm activities**

**(Takamasa et al.: How landscape factors relate to biodiversity-economic performance in an Estonian grassland-rich region)**

| **Land use category** | **Modelled grass type/crop name** | **Modelled management option** |
| --- | --- | --- |
| Semi-natural grassland | Boreal Baltic coastal meadows | Grazing |
|  | Boreal Baltic coastal meadows | Mulching |
|  | Nordic alvar and precambrian calcareous flatrocks | Grazing |
|  | Nordic alvar and precambrian calcareous flatrocks | Mulching |
|  | Fennoscandian wooded pastures | Mowing- 1 cut per year for hay |
|  | Fennoscandian wooded pastures | Mulching |
|  | Northern boreal alluvial meadows | Grazing |
|  | Northern boreal alluvial meadows | Mowing - 1 cut per year for hay |
|  | Northern boreal alluvial meadows | Mulching |
|  | Alkaline fens | Grazing |
|  | Lowland hay meadows | Mowing - 1 cut per year for hay |
|  | Lowland hay meadows | Mulching |
|  | Fennoscandian lowland species-rich dry to mesic grasslands | Mowing - 1 cut per year for hay |
|  | Fennoscandian lowland species-rich dry to mesic grasslands | Mulching |
| Permanent grassland | Permanent grass | Grazing |
|  | Permanent grass | Mowing - 1 cut per year for hay |
|  | Permanent grass | Mowing - 2 cuts per year for hay |
|  | Permanent grass | Mowing - 3 cuts per year for hay |
|  | Permanent grass | Mowing for hay, then grazing |
|  | Permanent grass | Mowing for silage, then grazing |
|  | Permanent grass | Mulching |
| Arable land | Field grass | Silage production |
|  | Field grass | Baled silage production |
|  | Fallow land |  |
|  | Field beans | Ploughing |
|  | Field beans | Reduced ploughing |
|  | Field beans | Direct seeding |
|  | Field peas | Ploughing |
|  | Field peas | Reduced ploughing |
|  | Field peas | Direct seeding |
|  | Green maize | Ploughing |
|  | Oats | Ploughing |
|  | Oats | Reduced ploughing |
|  | Oats | Direct seeding |
|  | Rye | Ploughing |
|  | Rye | Reduced ploughing |
|  | Rye | Direct seeding |
|  | Spring barley | Ploughing |
|  | Spring barley | Reduced ploughing |
|  | Spring barley | Direct seeding |
|  | Spring wheat | Ploughing |
|  | Spring wheat | Reduced ploughing |
|  | Spring wheat | Direct seeding |
|  | Winter wheat | Ploughing |
|  | Winter wheat | Reduced ploughing |
|  | Winter wheat | Direct seeding |
|  | Buckwheat | Ploughing |
|  | Buckwheat | Reduced ploughing |
|  | Buckwheat | Direct seeding |
|  | Potatoes | Ploughing |
|  | Spring rapeseed | Ploughing |
|  | Winter rapeseed | Ploughing |
